# Improving Grapevine Heat Stress Resilience with Marine Plant Growth-Promoting Rhizobacteria Consortia

**DOI:** 10.3390/microorganisms11040856

**Published:** 2023-03-27

**Authors:** João Carreiras, Ana Cruz-Silva, Bruno Fonseca, Ricardo C. Carvalho, Jorge P. Cunha, João Proença Pereira, Catarina Paiva-Silva, Soraia A. Santos, Rodrigo Janeiro Sequeira, Enrique Mateos-Naranjo, Ignacio D. Rodríguez-Llorente, Eloísa Pajuelo, Susana Redondo-Gómez, Ana Rita Matos, Jennifer Mesa-Marín, Andreia Figueiredo, Bernardo Duarte

**Affiliations:** 1 MARE—Marine and Environmental Sciences Centre & ARNET–Aquatic Research Network Associated Laboratory, Faculdade de Ciências da Universidade de Lisboa, Campo Grande, 1749-016 Lisbon, Portugal; amcsilva@fc.ul.pt (A.C.-S.); rfcruz@fc.ul.pt (R.C.C.); 2 Plant Functional Genomics Group, Departamento de Biologia Vegetal, BioISI—Biosystems and Integrative Sciences Institute, Faculdade de Ciências da Universidade de Lisboa, Campo Grande, 1749-016 Lisboa, Portugalaafigueiredo@ciencias.ulisboa.pt (A.F.); 3 Grapevine Pathogen Systems Lab, BioISI, Faculdade de Ciências da Universidade de Lisboa, Campo Grande, 1749-016 Lisbon, Portugal; 4 Estação Vitivinícola Nacional, 2565-191 Dois Portos, Portugal; 5 Department of Plant Biology and Ecology, Faculty of Biology, University of Seville, 41012 Seville, Spainjmesam@us.es (J.M.-M.); 6 Department of Microbiology and Parasitology, Faculty of Pharmacy, University of Seville, 41012 Seville, Spain; 7 Departamento de Biologia Vegetal, Faculdade de Ciências da Universidade de Lisboa, Campo Grande, 1749-016 Lisbon, Portugal

**Keywords:** *Vitis vinifera*, heatwave stress, bioaugmentation, stress physiology, PGPB, osmotic stress, root inoculation, halotolerant bacteria

## Abstract

Amid climate change, heatwave events are expected to increase in frequency and severity. As a result, yield losses in viticulture due to heatwave stress have increased over the years. As one of the most important crops in the world, an eco-friendly stress mitigation strategy is greatly needed. The present work aims to evaluate the physiological fitness improvement by two marine plant growth-promoting rhizobacteria consortia in *Vitis vinifera* cv. Antão Vaz under heatwave conditions. To assess the potential biophysical and biochemical thermal stress feedback amelioration, photochemical traits, pigment and fatty acid profiles, and osmotic and oxidative stress biomarkers were analysed. Bioaugmented grapevines exposed to heatwave stress presented a significantly enhanced photoprotection capability and higher thermo-stability, exhibiting a significantly lower dissipation energy flux than the non-inoculated plants. Additionally, one of the rhizobacterial consortia tested improved light-harvesting capabilities by increasing reaction centre availability and preserving photosynthetic efficiency. Rhizobacteria inoculation expressed an osmoprotectant promotion, revealed by the lower osmolyte concentration while maintaining leaf turgidity. Improved antioxidant mechanisms and membrane stability resulted in lowered lipid peroxidation product formation when compared to non-inoculated plants. Although the consortia were found to differ significantly in their effectiveness, these findings demonstrate that bioaugmentation induced significant heatwave stress tolerance and mitigation. This study revealed the promising usage of marine PGPR consortia to promote plant fitness and minimize heatwave impacts in grapevines.

## 1. Introduction

Grapevines are one of the most economically important fruit crops in the world, with over 74.8 million tonnes of grapes produced worldwide in 2021 [1]. Climate change poses a significant threat to the viticulture industry, with rising temperatures leading to increased heat stress and consequently drought stress [2]. Temperature severely influences the grapevine’s main physiological processes, development, quality and productivity [3]. A 10 °C base temperature is required for the onset of the grapevine’s vegetative cycle; it is also known that if the high-temperature threshold peaks at critical points of development, negative impacts occur [4], namely on photosynthesis [5], berry size, sugar accumulation and ripening [6].

Due to climate change, the impact of high temperatures and heatwaves (HW) will likely be a major concern for winegrowers in the upcoming decades. A heatwave is defined by the World Meteorological Organization as a prolonged episode (5 days or longer) in which the daily maximum temperature is higher than the average maximum temperature by 5 °C [7]. High temperatures during heatwaves are usually accompanied by a decrease in soil moisture, leading to a decrease in plant water content [8]. It has been pointed out that these extreme weather events will be more frequent and with an increased duration in the next decades [9]. In 2019, three consecutive heatwave events occurred in Europe with temperatures reaching 44 °C in some regions during June [10]. Moreover, previous studies indicate that HW impacts vary among plant families [11,12]; they can be species-specific [13], depend on the plant’s life history [14] and vary from photochemical to biochemical [12,13] to alterations in plant gene expression [11]. Nowadays, viticulture is adjusting to this new reality through more effective management methodologies, such as irrigation, fruit size manipulation and variety selection, but these practices are not ideal and restrict the exploration of wine-growing varieties [15]. Thus, considering the economic and cultural importance of grapevines, it is of utmost importance to address the effects of these extreme events and to develop strategies to minimize their impacts.

Although agricultural systems often subject soil microbes to negative selection pressure, in the last few years new regenerative agriculture practices are being applied and awareness about the importance of microbial diversity is rising [16]. The potential of plant growth-promoting rhizobacteria (PGPR) is gaining attention for bolstering sustainable agriculture while ensuring agricultural productivity and for combating soil deterioration caused by the use of synthetic agrochemicals. PGPR–based solutions are environmental-friendly, as they are natural sources of renewable nutrients required for maintaining soil health and biology [17,18]. In fact, several strains have already been commercially used as efficient biofertilizers, while also appearing to be cost-effective [19]. Plant growth-promoting bacteria are microorganisms that have a positive impact on plant growth and development. These bacteria can enhance plant growth and stress tolerance through the production of phytohormones, such as indole acetic acid (IAA), cytokinin, abscisic acid and the reduction of ethylene [17,20,21]. Additionally, PGPR can also provide plants with abiotic stress resistance or tolerance and, although relatively unexplored, recent findings highlight heat stress amelioration and their promising practical applications [21,22]. Microbial fertilizers may combine different strains to promote multiple functions aiding plant growth-promoting activity; however, these solutions are not universal as different habitats and cultivation strategies largely influence specific PGPR communities [23]. Nevertheless, there is a need to identify new PGPR resources to cope with different challenges. Salt marshes are unique ecosystems that are exposed to high salinity and fluctuations in water levels, making them a potential source of PGPR with stress tolerance properties [24]. The bioaugmentation of plants with these marine PGPR has been shown to aid the plants in resisting and overcoming several abiotic challenges from nutrient deprivation [25] and osmotic stress [26,27], including thermal and extreme stress [28,29]. Moreover, a single strain microorganism usage does not allow the activation of all possible growth-promoting mechanisms; thus, in order to elicit all potential traits, the application of PGPR consortia is increasingly more common in agricultural practices and is of current interest in research [28,30]. 

In this study, compatible marine rhizobacteria isolated from salt marshes were selected to build two potentially effective PGPR consortia to allow for multiple simultaneous PGP property expressions. The first is composed of *Pseudomonas composti*, *Bacillus zhangzhouensis* and *Pseudarthrobacter oxydans*; the second is composed of *Aeromonas aquariorum*, *Bacillus methylotrophicus* and *Bacillus aryabhattai*. Bacterial strains were chosen for their great potential as bioinoculants and grouped for their different complementary activities and traits (as seen in Table 1 and references therein) with no conflicting effect between them. In the present study, we aim to evaluate the PGPR bioaugmentation impact on the physiological fitness of a *Vitis vinifera* heat susceptible cultivar, namely Antão Vaz [31], when exposed to HW simulation. This will be evaluated by addressing leaf thermography, photochemical performance, osmotic regulation mechanisms, pigment composition, antioxidant defences and fatty acid profiles, thus providing deeper insight into the mechanisms underlying PGPR stimulation of grapevine tolerance to HW stress.

## 2. Materials and Methods

### 2.1. Plant and Growing Conditions

*Vitis vinifera* cv. “Antão Vaz” in the rootstock 1103P wood cuttings were bought from the VitisOeste Nursery (Portugal). Plants were supplied without roots. All the sanitary conditions were assured. Grapevine wood cuttings were planted in pots containing soil from the INIAV Dois Portos vineyard (Colecção Ampelográfica Nacional, CAN). CAN is property of INIAV-Estação Vitivinícola Nacional (Dois Portos), located at Quinta da Almoinha, 60 km north of Lisbon (9°11′19″ W; 39°02′31″ N; 75 m above sea level). The soil from the ampelographic field vineyard was characterized by a high percentage of clay (40.6%), a of pH 8.3, low organic matter (1.25%) and suitable nutrient content. It was chosen to mimic to the extent possible the grapevine environment in realistic cultivation schemes [32]. Wood cuttings (40 cm with an average of three nodes) were then grown under greenhouse conditions, i.e., a natural day/night rhythm with temperatures of 25 °C day/21 °C night, according to previously optimized conditions [33]. Plants were kept under these conditions for three months to acclimate to the new environment and allow for root and leaf biomass development.

### 2.2. Rhizobacteria Used for Inoculation in This Study

Plant growth-promoting bacterial consortia (Table 1) used in this experiment were made with rhizobacteria that were isolated, identified and described in previous studies. Bacteria isolates were obtained from different halophytes inhabiting southwestern Spain coastal salt marshes: *P. composti* SDT3 and *A. aquariorum* SDT13 were collected from *Spartina densiflora* rhizosphere at Tinto estuary (37°13′ N 6°54′ W); *B. methylotrophicus* SMT38 and *B. aryabhattai* SMT48 from *Spartina maritima* rhizosphere at Odiel estuary (37°13′ N 6°57′ W); *B. zhangzhouensis* HPJ40 and *P. oxydans* SRT15 were isolated from *Halimione portulacoides* and *Salicornia ramosissima* rhizospheres, respectively, at Piedras estuary (37°16′ N 7°09′ W).

**Table 1:** Consortia design and plant growth-promoting rhizobacterial (PGPR) trait summary.

Consortium Number	Bacterial Strains	Biofilm Production	P-Solubilization (mm halo)	Siderophore Production (mm halo)	ACC Deaminase (µmoles α-cetog/h/mg Protein)	IAA Production (mg/mL)	N-Fixation	Reference
C1	*Pseudomonas composti* SDT3	−	+	32	−	−	−	[34]
	*Bacillus zhangzhouensis* HPJ40	+	11	15	−	−	+	[35]
	*Pseudarthrobacter oxydans* SRT15	−	9	−	−	20.99	+	
C2	*Aeromonas aquariorum* SDT13	−	+	15	−	3.40	−	[34]
	*Bacillus methylotrophicus* SMT38	+	−	10	−	−	+	[36]
	*Bacillus aryabhattai* SMT48	−	2.5	7	−	3.25	+	

+ positive; − negative.

### 2.3. Preparation of Bacterial Inoculants

To prepare the six suspensions for root inoculation, rhizobacteria were grown separately in 250 mL Erlenmeyer flasks containing 50 mL of tryptic soy broth (TSB) medium in a rotary shaker (150 rpm, 30 °C) for 24 h. The cultures were then centrifugated in 50 mL sterile Falcon tubes at 5000× *g* for 10 min at room temperature, and the supernatant was discarded. Pellets were washed twice with sterile physiological saline solution (NaCl 0.9% *w/v*) (by resuspension and centrifugation) and adjusted to a final concentration of 10^8^ CFU mL^−1^ (OD600 = 1) for each bacteria [35]. Bacterial suspensions were then adequately mixed in equal amounts to produce the two final inoculant suspensions. To assess the potential incompatibility effects of the bacteria species, isolates were plated together in a solid agar TSB medium, and their growth halo diameter was compared with the growth halo of each species when plated individually. No significant differences were observed between the co-cultures and the monocultures, indicating no incompatibility between the bacteria. 

### 2.4. Experimental Setup and Root Inoculation

**Figure 1:**
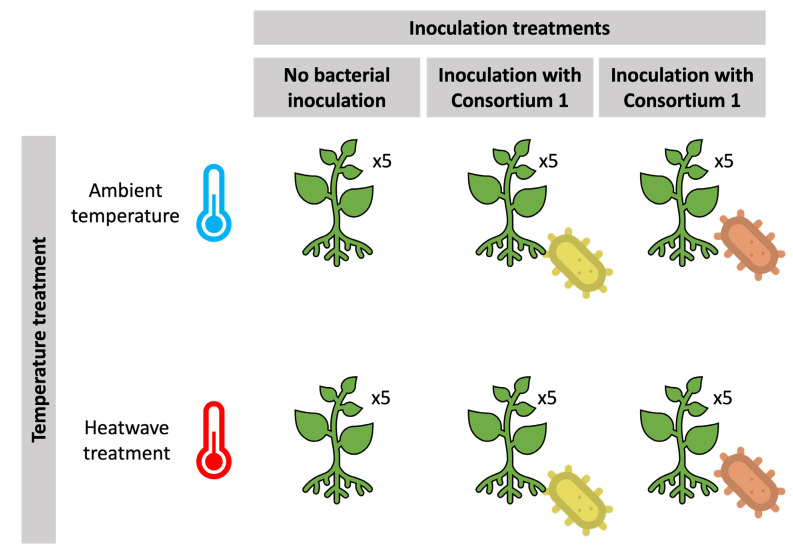
Experimental design of the thermal and inoculation treatments applied.

Following the acclimation period, plants were divided into three groups for root inoculation. Each pot of the inoculated groups was watered with 50 mL of the respective and above-mentioned consortia solution and diluted with distilled water to achieve a concentration of 10^7^ CFU mL^−1^. Each pot in the non-inoculated group was watered with 50 mL of distilled water. This procedure was completed three times: 45, 15 and 7 days before the experiment started. After inoculation, *V. vinifera* individuals from each group were divided and exposed to two thermal treatments: control (25/21 °C day/night) and heatwave treatment (42/38 °C day/night) for 5 days, as seen in the experimental design (Figure 1). All treatments consisted of 5 replicate plants. Heatwave simulation was performed following a widely used and established definition found in the literature [37,38] and, as employed in previous studies [11,14], in controlled-environment chambers (Aralab/Fitoclima 18.000 EH, Lisbon, Portugal) with a light intensity of 300 μmol m^−2^ s^−1^, 70 ± 5% relative humidity and watered every two days with distilled water. 

Following the end of the trials, infrared thermography and chlorophyll fluorescence measurements were performed; consecutively, the plants were harvested. Leaf samples for biochemical measurements were immediately flash-frozen in liquid-N_2_ and stored at −80 °C until analysis.

### 2.5. Leaf Infrared Thermography

At the end of the exposure trials, thermal images were obtained with a FLIR E50bx infrared camera (FLIR Systems, Inc., Wilsonville, OR, USA), producing images of 320 × 240 resolution with an accuracy of ±0.045 °C. Ten leaves were randomly selected from plants of both treatments; a water container at room temperature was kept near the leaves as a reference. The average temperature of each leaf was calculated on each image. All image processing and analysis were performed in FLIR Tools software (version 6.4.18039.1003, FLIR Systems Inc., Wilsonville, OR, USA).

### 2.6. Imaging Pulse Amplitude Modulated Fluorometry (iPAM)

After infrared thermography, imaging pulse amplitude modulated (iPAM) chlorophyll fluorescence measurements were performed using a customized open FluorCam imaging system (FC 800-D/3535-FAST; Photo System Instruments, Brno, Czech Republic) on 30 min dark-adapted plants. In addition, the OJIP transient (or Kautsky curves) measurements were performed to evaluate the plant’s photochemical apparatus functioning through the chlorophyll fluorescence induction kinetics of the PS II. This was achieved by irradiating a dark-adapted plant with a saturating light intensity of 3500 µmol m^−2^ s^−1^, leading to the production of a polyphasic rise in fluorescence (OJIP). Level O represents all the open reaction centres at the onset of illumination with no reduction of Quinone A (Q_A_) (fluorescence intensity lasts for 10 ms). The O to J transient indicates the net photochemical reduction of Q_A_ (the stable primary electron acceptor of PS II) to Q_A_^−^ (lasts for 2 ms). The J to I transition is due to all reduced states of closed RCs such as Q_A_^−^ Q_B_^−^, Q_A_ Q_B_^2−^ and Q_A_^−^ Q_B_ H_2_ (lasts for 2–30 ms). The P-step coincides with a maximum concentration of Q_A_^−^ Q_B_^2^ with the plastoquinol pool maximally reduced; it also reflects a balance between the light incident at the PS II side and the rate of utilization of the chemical (potential) energy, as well as the rate of heat dissipation. Supplementary Table S1 summarizes all the parameters calculated from the fluorometric analysis [39]. 

### 2.7. Pigment Profiling

Samples from the differently treated test groups were weighted before (FW) and after (DW) freeze-drying; these data were used to calculate the leaf water content as follows:

(1) $$WC(\%)=\frac{\left(FW-DW\right)}{FW}$$

Freeze-dried leaves (approximately 200 mg) were extracted with 6 mL pure acetone, subjected to a 2 min ultra-sound bath to ensure complete cell disruption and extracted in the dark at −20 °C for 24 h. After extraction, samples were centrifuged at 4.000× *g* for 15 min at 4 °C. The supernatants were scanned from 350 nm to 750 nm in 1 nm steps using a Shimadzu UV/VIS UV1601 dual-beam spectrophotometer (Shimadzu Corporation, Kyoto Japan). The sample absorbance spectra were analysed employing the Gauss peak spectra (GPS) method fitting library, using SigmaPlot Systat 13.2 Software (Systat Software Inc., San Jose, CA, USA) [40]. This method allowed pigment recognition and quantification from the sample absorbance spectrum, ascertaining the leaf pigment profile, chlorophyll *a*, chlorophyll *b*, auroxanthin, antheraxanthin, β-carotene, lutein, violaxanthin and zeaxanthin. For a better evaluation of the light-harvesting and photoprotection mechanisms, the de-epoxidation state (DES) was calculated as follows:

(2) $$DES=\frac{\left(\left[Antheraxanthin\right]+\left[Zeaxanthin\right]\right)}{\left(\left[Violaxanthin\right]+\left[Antheraxanthin\right]+\left[Zeaxanthin\right]\right)}$$

### 2.8. Proline Content

Leaf proline content was assessed according to [41]. Plant material was homogenized in sulfosalicylic acid 3% (*w/v*). After extraction, samples were centrifuged at 10,000 rpm for 15 min at 4 °C. The supernatant (2 mL) was added with 2 mL glacial acetic acid and 2 mL acid ninhydrin and incubated for 1 h at 100 °C in a dry bath; after this, the reaction was stopped in an ice bath. After cooling, 4 mL of toluene was added to the reaction mixture and vortexed to ensure the complete mixing of the organic and aqueous phases. The absorbance of the organic phase was analysed at 520 nm and compared with a standard curve of proline and expressed in µmol g^−1^ FW.

### 2.9. Oxidative Stress Biomarkers

Enzyme extracts were produced by grinding a 250 mg leaf sample with 4 mL of a 50 mM sodium phosphate buffer (pH 7.6) supplemented with 0.1 mM Na-EDTA at 4 °C [42]. The produced homogenate was centrifuged at 8890× *g* for 20 min at 4 °C, and the supernatant was maintained on ice for enzymatic and protein analysis. 

The enzyme activity measurements of catalase (CAT, EC.1.11.1.6.), ascorbate peroxidase (APx, E.C. 1.11.1.11), guaiacol peroxidase (GPX, E.C. 1.11.1.7) and superoxide dismutase (SOD, E.C. 1.15.1.1) were performed in a dual-beam spectrophotometer (Shimadzu UV/VIS UV1601 Spectrophotometer) using quartz cuvettes. According to [43], catalase activity was assayed through the evaluation of H_2_O_2_ consumption by an absorbance decline at 240 nm (ε = 39.4 mM^−1^ cm^−1^). Ascorbate peroxidase activity was analysed according to [42] by monitoring the ascorbate oxidation, using the absorbance decline at 290 nm (ε = 2.8 mM^−1^ cm^−1^). Guaiacol peroxidase measurement was performed according to [44] by monitoring the absorbance rise at 470 nm corresponding to the generation of guaiacol oxidation products (ε = 26.6 mM^−1^ cm^−1^). Superoxide dismutase activity was measured according to [45] by calculating the oxidation rate of pyrogallol using the absorbance at 325 nm. Protein quantification was performed according to [46]. Lastly, an oxidative ratio was calculated considering the intricate reactions occurring between SOD and the peroxidase enzymes. The oxidative ratio can reflect the balance between this hydrogen peroxide production and consumption and was calculated according to the following equation:

(3) $$Oxidative\;ratio=\frac{SOD}{CAT+APx+GPx}$$

Membrane lipid peroxidation in plant samples was evaluated through the quantification of thiobarbituric acid reactive substances (TBARS) according to [47]. Samples (100 mg) were homogenized in a 2 mL solution of fresh 0.5% (*w/v*) thiobarbituric acid (TBA) and 20% (*w/v*) trichloroacetic acid (TCA) solution and extracted for 30 min at 95 °C in a dry bath. After this period, the reaction was stopped on ice, and samples were centrifuged at 4.000× *g* for 5 min at 4 °C. The absorbance of the supernatant was read at 532 nm and 600 nm in a Shimadzu UV/VIS UV1601 spectrophotometer. Sample TBARS concentration was calculated according to the following equation, using the malondialdehyde (MDA) molar extinction coefficient (ε = 155 mM^−1^ cm^−1^):

(4) $$A_{532nm}-A_{600nm}=\left[MDA\right]\;mM\times \varepsilon MDA$$

### 2.10. Fatty Acid Profiles

Plant sample fatty acid analysis was performed according to [48] by direct trans-esterification of the leaf samples in freshly prepared methanol sulfuric acid (97.5:2.5, *v/v*) for 1 h at 70 °C. As an internal standard, heptadecanoic acid (C17:0) was added to the extraction medium. Fatty acid methyl esters (FAMEs) were recovered using petroleum ether, dried under an N_2_ stream and re-suspended in hexane. Reconstituted FAMEs were analysed through gas chromatography (Varian 430-GC gas chromatograph, Varian Inc., Palo Alto, CA, USA), equipped with a hydrogen flame ionization detector set at 300 °C, by injecting 1 µL of the FAME solution (injector temperature = 270 °C; split ratio = 50). FAMEs were separated using a fused-silica capillary column (50 m × 0.25 mm; WCOT Fused Silica, CP-Sil 88 for FAME; Varian), maintained at a constant nitrogen flow of 2 mL min^−1^ and the column oven set to 190 °C. Fatty acid identification was performed by comparison of retention times with standards (Sigma-Aldrich) and chromatograms analysed by the peak surface method using the Galaxy software. To determine membrane saturation levels, the double bond index (DBI) was calculated according to the following equation:

(5) $$DBI=\frac{2\times \left(\%monoenes+2\times \%dienes+3\times \%trienes+4\times \%tetraenes+5\times \%pentaenes\right)}{100}$$

### 2.11. Statistical Analysis

Boxplots with probability density of the data at different values smoothed by a kernel density estimator were computed and plotted using the *ggplot2* package in R-Studio Version 1.4.1717 (RStudio Inc., Boston, MA, USA). Non-parametric Kruskal–Wallis with Bonferroni post hoc tests for variable comparison between inoculation and thermal treatments were performed in R-Studio Version 1.4.1717 using the *agricolae* package [49]. Canonical analysis of principal coordinates (CAP) was used to evaluate the ability to classify individuals successfully according to the thermal and inoculation treatments applied using each of the considered biochemical and biophysical traits, utilizing Primer 6 software (Primer-E Ltd., Devon, UK) [50].

## 3. Results

### 3.1. Thermography

**Figure 2:**
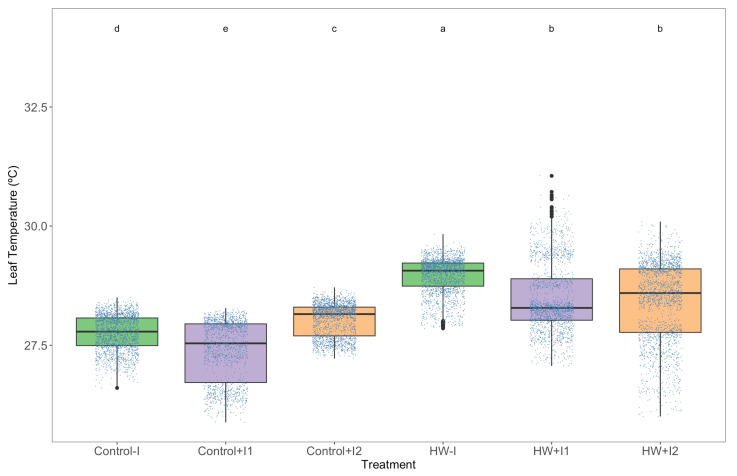
Leaf surface temperature measured through infrared thermography of non-inoculated (−I) and marine PGPR consortia 1- and 2-inoculated (+I) *Vitis vinifera* plants, under normal (control) thermal regimes and exposed to the heatwave (HW) (average ± standard deviation, N = 5, letter denotes significant differences at *p* < 0.05).

Grapevine exposure to the heatwave significantly increased their leaves’ surface temperature (Figure 2). Nonetheless, infrared thermography measurements showed significant variation within thermal conditions as well. Compared to the control non-inoculated plants, C1- and C2-inoculated plants exhibited significantly lower and higher leaf temperatures, respectively. At the same time, the HW-exposed plants’ leaf temperature increase was less pronounced in the leaves of the PGPR-inoculated plants.

### 3.2. Photochemical Processes

**Figure 3:**
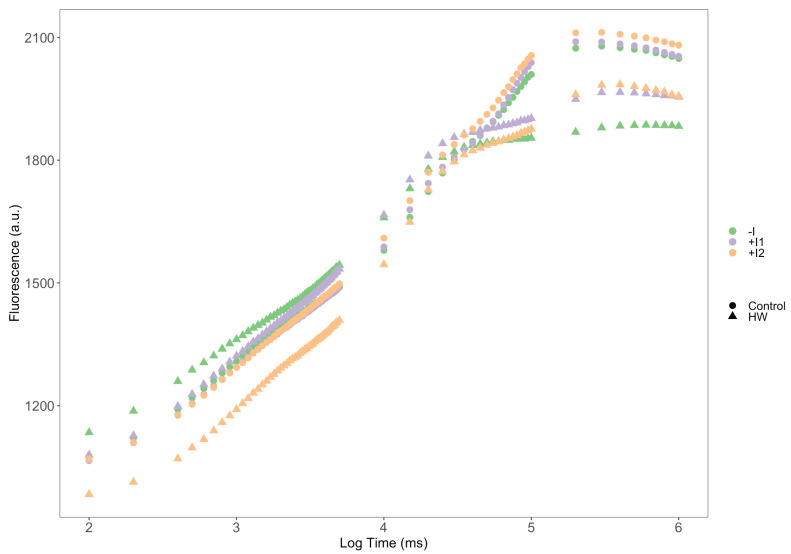
Kautsky fluorescence induction curves of non-inoculated (−I) and marine PGPR consortia 1- and 2-inoculated (+I) *Vitis vinifera* dark-adapted leaves, under normal (control) thermal regimes and exposed to heatwave (HW) (N = 5, average).

The Kautsky plots (Figure 3) from the in vivo iPAM fluorometric analysis showed a contrast between the thermal treatments’ observable fluorescence induction curves. Plants exposed to control temperature from all three groups presented very similar and high fluorescence levels with typical inflection points of standard OJIP curves. In comparison, lower fluorescence values were evidenced in the HW-exposed plants. Within the HW-treated groups, a higher relative fluorescence variation was seen, evident in the K-step (300 ms) dissociation, observably higher in the non-inoculated plants and lower in the C2-inoculated plants. Non-inoculated plants presented lower maximum fluorescence. 

**Figure 4:**
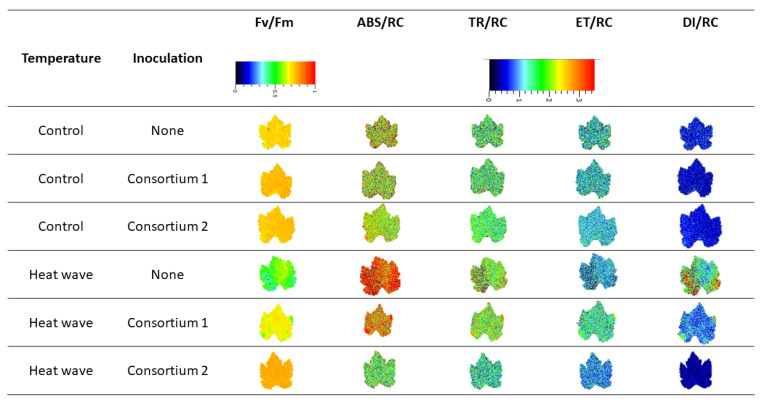
Imaging pulse amplitude modulated (iPAM) fluorescence derived parameter images: PS II quantum yield (F_v_/F_m_), absorbed (ABS/RC), trapped (TR_0_/RC), transported (ET_0_/RC) and dissipated (DI_0_/RC) energy fluxes on a reaction centre basis relative to dark-adapted non-inoculated and marine PGPR consortia 1- and 2-inoculated *Vitis vinifera* leaves, under normal (control) thermal regimes and exposed to the heatwave.

The fluorescence images obtained from the dark-adapted leaves of tested plants can reveal the PGPR implication in the experimental heatwave event (Figure 4). Under normal temperature conditions, the observed parameters were shown to be relatively similar between the different plant groups. However, under heatwave treatment, differences were exacerbated between plant inoculation treatments. When compared to control condition grapevines, HW-exposure was found to be lower in PS II quantum yield (F_v_/F_m_) and higher in the dissipated (DI_0_/RC) and absorbed (ABS/RC) energy fluxes in both non-inoculated and, less so, in C1-inoculated individuals. In overall imaging PAM-derived parameters, C2-inoculated plants under heatwave treatment appeared identical to their control counterpart. 

**Figure 5:**
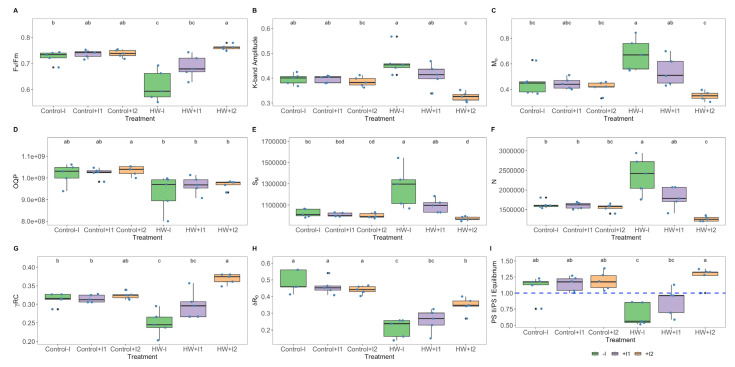
OJIP derived parameters: (**A**) PSII quantum yield (F_v_/F_m_), (**B**) K-band amplitude, (**C**) net rate of PS II reaction centre closure (M_0_), (**D**) size of the oxidized quinone pool (QOP), (**E**) the energy needed to close all reaction centres (S_M_), (**F**) total number of electrons transferred into the electron transport chain (N), (**G**) the probability that a PSII chlorophyll molecule functions as an RC (γRC), (**H**) PS I efficiency in reducing its electron acceptors (δR_0_), (**I**) PS II/PS I Equilibrium in non-inoculated (−I) and marine PGPR consortia 1- and 2-inoculated (+I) *Vitis vinifera* dark-adapted leaves, under normal (control) thermal regimes and exposed to the heatwave (HW) (average ± standard deviation, N = 5, letter denotes significant differences at *p* < 0.05).

Analysing the extracted OJIP parameters, several significant variations were detected in the PS II and PS I photochemical traits among thermal treatments and between PGPR-treated plants. The non-inoculated plants’ photosystem II quantum yield (F_v_/F_m_; Figure 5A) and PS I efficiency in reducing electron acceptors (δR_0;_ Figure 5G) were significantly lower in HW conditions than at normal temperature conditions. In comparison, no significant differences between both PGPR-inoculated groups could be found. Nonetheless, C2-inoculated plants exhibited significantly higher values of both these parameters within heatwave conditions. Similarly, the reverse pattern was observed in the K-band amplitude, the net rate of PS II reaction centre closure (M_0_), the energy needed to close all reaction centres (S_M_) and the total number of electrons transferred into the electron transport chain (N) (Figure 5B,C,E,F). Non-inoculated plants exposed to heatwave simulation presented significantly higher values in these parameters (M_0_, S_M_ and N) than their control counterpart, whereas C2-inoculated exhibited a significantly lower K-band amplitude. In contrast, C1-inoculated plants did not show any significant difference between thermal conditions. The size of the oxidized quinone pool (QOP, Figure 5D) was significantly lower in C2-bioaugmented plants under heat stress than at control conditions. The photosystem I efficiency in reducing electron acceptors (δR_0_, Figure 5H) showed a significant reduction in all HW-exposed groups, when compared to the corresponding control; this was more significant in the non-inoculated and lower in C2-inoculated individuals. Furthermore, in HW conditions, the equilibrium constant of the redox reactions between PS II and PS (Figure 5I) was observed to be significantly lower in the non-inoculated and C1-inoculated plants than in their control counterparts. However, C2-inoculated grapevines maintained this parameter at control levels.

**Figure 6:**
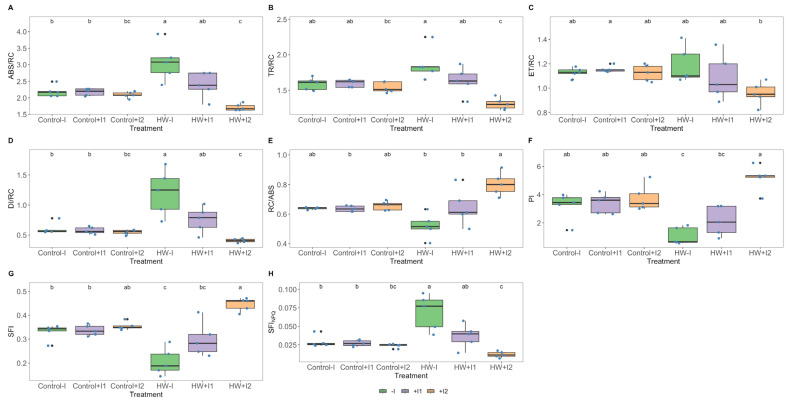
Reaction centre-based energetic parameters: (**A**) absorbed (ABS/RC), (**B**) trapped (TR/RC), (**C**) electron transport (ET/RC), (**D**) dissipation (DI/RC) energy fluxes by reaction centre and (**E**) reaction centre density within the PS II antennae (RC/ABS), (**F**) performance index (PI), (**G**) structural and functional index for photosynthesis (SFI), (**H**) non-photosynthetic or dissipation structural and functional index (SFI_NPQ_) in non-inoculated (−I) and marine PGPR consortia 1- and 2-inoculated (+I) *Vitis vinifera* dark-adapted leaves, under normal (control) thermal regimes and exposed to the heatwave (HW) (average ± standard deviation, N = 5, letter denotes significant differences at *p* < 0.05).

The statistical analysis of the reaction centre-based energy fluxes revealed a significant impact from rhizobacteria inoculation and marked trends in the heatwave conditions, as previously observed (Figure 3). Compared to the control, energy flux differences in heatwave conditions were noted in all tested groups. Heatwave-exposed non-inoculated plants presented significantly higher absorbed (ABS/RC; Figure 6A), trapped (TR/RC; Figure 6B) and dissipated (DI/RC; Figure 6D) energy fluxes. C1-inoculated plants also followed the same trend, yet variation was non-significant. In addition, inoculated plants exhibited a reduction in their electron transport energy flux (ET/RC; Figure 6C) following HW exposure. Reaction centre II density within the antenna chlorophyll bed of PS II (RC/ABS; Figure 6E) was found to be significantly higher in the C2-inoculated plants under heatwave treatment when compared to the remaining heatwave exposed groups. Heatwave exposure resulted in significantly lower performance index (PI, Figure 6F) and structural and functional index (SFI, Figure 6G) values in non-inoculated plants, whereas bioaugmented plants showed similar values to the corresponding control. As expected, a mirror image of SFI was found in the dissipation structural and functional index (SFI_NPQ_, Figure 6H). It is noteworthy that HW-exposed non-inoculated and C2-inoculated plants revealed significant differences between them in all these parameters. 

### 3.3. Leaf Pigment Profile

Observing grapevine leaf pigment concentrations and ratios (Table 2) at control temperature, no significant differences between groups were detected. Heatwave-exposed groups showed a higher Chl *a*, chlorophyll *b* (Chl *b*), total chlorophyll (TChl), total carotenoid (Tcarot) and zeaxanthin (Zea) than their corresponding control; this was significant in C1-inoculated plants. The pheophytin *a* (Pheo *a*), β-carotenoids (βcarot), antheraxanthin (Anthe) and lutein concentrations, were not seen to differ with temperature or inoculation treatments. The de-epoxidation state (DES) did not reveal any impact from temperature or inoculation treatments. The chlorophyll *a*/*b* ratio (Chl *a/b*), in HW-exposed C1-inoculated plants was shown to be lower than its corresponding control group. Similarly, the total carotenoid to total chlorophyll (TCar/TChl) ratio was found to be significantly lower in heatwave-treated C2-inoculated plants. Additionally, no significant differences were observed in the chlorophyll degradation index (CDI).

### 3.4. Water and Proline Quantification

**Figure 7:**
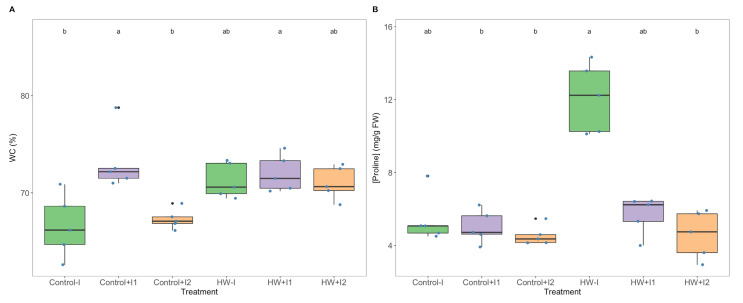
Leaves (**A**) water content (WC) and (**B**) proline concentrations of non-inoculated (−I) and marine PGPR consortia 1- and 2-inoculated (+I) *Vitis vinifera*, under normal (control) thermal regimes and exposed to the heatwave (HW) (average ± standard deviation, N = 5, letter denotes significant differences at *p* < 0.05).

Grapevine leaf water content (WC; Figure 7A) showed no significant differences between the thermal treatments. However, at control conditions, WC was found to be significantly higher in the C1-inoculated plants. Proline content (Figure 7B) in both PGPR- bioaugmented groups under heat stress showed values similar to those observed in the corresponding control. Additionally, at heatwave conditions, non-inoculated grapevines exhibited a higher proline concentration; this difference is significant when compared to C2-inoculated plants. 

### 3.5. Antioxidant Enzymatic Activities

**Figure 8:**
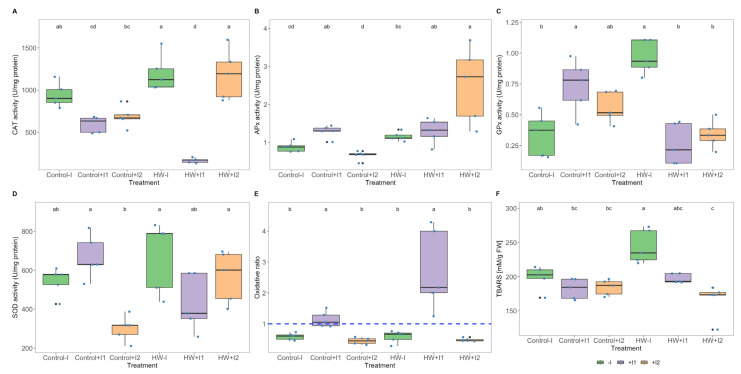
Oxidative stress biomarkers, (**A**) catalase (CAT), (**B**) ascorbate peroxidase (APx), (**C**) guaiacol peroxidase (GPx), (**D**) superoxide dismutase (SOD) activities (U mg^−1^ protein), (**E**) oxidative ratio and (**F**) thiobarbituric acid reactive substances (TBARS; µM MDA g^−1^ FW) in non-inoculated (−I) and marine PGPR consortia 1- and 2-inoculated (+I) *Vitis vinifera* leaves, under normal (control) thermal regimes and exposed to the heatwave (HW) (average ± standard deviation, N = 5, letter denote significant differences at *p* < 0.05).

Grapevine inoculation with marine PGPR consortia showed a significant impact on the leaf antioxidant enzyme activity under both thermal conditions. Catalase activity (Figure 8A), within heatwave thermal conditions was found to be significantly lower in the C1-inoculated plants. Ascorbate peroxidase (Figure 8B) activity exhibited thermal variation only in the C2-inoculated plants; it was significantly higher when HW-exposed. When compared to control groups, guaiacol peroxidase activity (Figure 8C) was found to be significantly higher and lower in HW-exposed non-inoculated and C1-inoculated plants, respectively. Moreover, under heatwave conditions, a significantly higher GPx was observed in the non-inoculated plants when compared to their inoculated counterparts. Superoxide dismutase activity (Figure 8D) thermal differences were found to be significant only in C2-inoculated plants, higher under heatwave treatment than in control conditions, and when compared to the other inoculates under control temperatures, exhibited a lower SOD activity. Observing the oxidative ratio (Figure 8E), which reflects enzymatic hydrogen peroxide production and consumption, C1-inoculated plants showed a significantly higher ratio in both control and heatwave treatments; even though significant differences were found among the non-inoculated and C2-inoculated antioxidant enzymatic, their oxidative ratio maintains the same balance between thermal conditions. Analysing the concentration of thiobarbituric acid reactive substances (Figure 8F), HW-exposed “non” and C1- and C2-inoculated plants presented, in that order, decremental TBARS concentrations which were significantly different between non-inoculated and C2-inoculated. 

### 3.6. Fatty Acid Profiles

**Figure 9:**
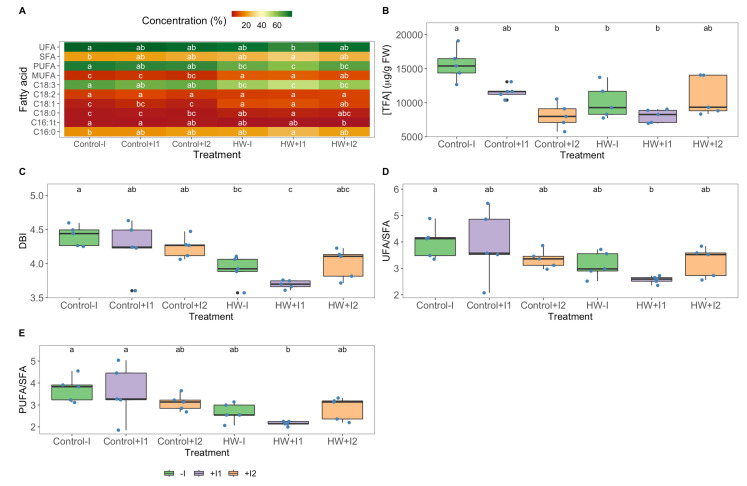
(**A**) Individual fatty acid and saturation class relative concentrations (monounsaturated fatty acids (MUFA)); polyunsaturated fatty acids (PUFA); saturated fatty acids (SFA); unsaturated fatty acids (UFA)); (**B**) total fatty acid content (TFA); (**C**) double bound index (DBI); (**D**) UFA/SFA ratio; (**E**) PUFA/SFA ratio in leaves of non-inoculated (−I) and marine PGPR consortia 1- and 2- inoculated (+I) *Vitis vinifera*, under normal (control) thermal regimes and exposed to heatwave (HW) (average ± standard deviation, N = 5, letter denotes significant differences at *p* < 0.05).

Analysis of total fatty acid composition in grapevine leaves revealed significant distinctions between groups and thermal conditions. Regarding fatty acid content (Figure 9A), no significant differences could be found in palmitic (C16:0), palmitoleic (C16:1t) and linoleic (C18:2) acids. When comparing experimental conditions, non-inoculated and C1-inoculated plants presented significantly higher stearic acid (C18:0) and significantly lower linolenic acid (C18:3) concentrations in heatwave conditions; in contrast, C2-inoculated plants showed no significative changes in these FAs. Moreover, the heatwave-treated groups were shown to have significantly lower oleic acid (C18:1) content. With respect to the unsaturated fatty acid (UFA) and saturated fatty acid (SFA) saturation classes, no meaningful differences between groups were found. Nonetheless, compared to control conditions, HW-treated non-inoculated and C1-inoculated plants exhibited significantly higher monounsaturated (MUFA) and significantly lower polyunsaturated (PUFA) fatty acids. However, no significant variation in saturation classes was seen in the C2-inoculated plants among thermal conditions. Regarding total fatty acid (TFA, Figure 9B) content, significance was only found in the non-inoculated plants, lower when exposed to heatwave treatment than at control conditions. Compared to their control groups, the non-inoculated and C1-inoculated *V. vinifera* double bound index (DBI; Figure 9C) was significantly lower when HW-exposed. The UFA/SFA and PUFA/SFA ratios (Figure 9D,E) presented no significant variation within thermal treatments and PGPR groups. 

### 3.7. Multivariate Physiological Profiles

**Figure 10:**
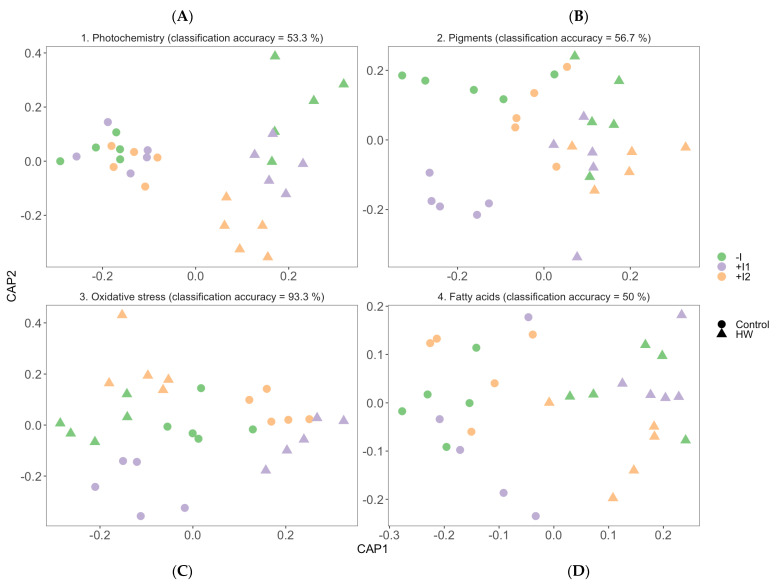
Canonical analysis of principal components (CAP) having as a basis, (**A**) the plant’s photochemistry, (**B**) pigment concentrations, (**C**) oxidative stress biomarker and (**D**) fatty acid concentration of non-inoculated (−I) and marine PGPR consortia 1- and 2-inoculated (+I) *Vitis vinifera* leaves, under normal (control) thermal regimes and exposed to heatwave (HW) (average ± standard deviation, N = 5, letter denotes significant differences at *p* < 0.05).

Canonical analysis of principal components (CAP) was performed to highlight and evaluate the impact of PGPR inoculation on grapevine physiological response to heatwave conditions (Figure 10). The multivariate approach to the Kautsky curve dataset (Figure 10A) identified the different thermal treatments and determined the separation of the PGPR-bioaugmented groups when exposed to heatwave conditions, especially noted in the C2-inoculated group isolation. Additionally, the observed photochemical similarities between the various plant groups under normal temperature conditions resulted in the low 53.3% correct classification efficiency. Regarding photosynthetic pigment CAP analyses (Figure 10B), a high overlap was observed between the various HW-treated plants evidenced by the low classification accuracy (56.7%). However, C1-inoculated plants under control conditions were found to be differentially grouped from the remaining samples. Similarly, in the leaf fatty acid profile CAP projection (Figure 10D), despite the 50% classification efficiency, thermal sorting was evident, and HW-exposed C2-inoculated plants were distinctively identifiable. The plots based on the oxidative stress dataset allowed for the description of all tested groups, revealing a highly efficient canonical classification of 93.3% for group allocation (Figure 10C). 

## 4. Discussion

Heatwave exposure led to a depression (both in fluorescence intensity and shape) in the Kautsky curves. This was more significant in non-inoculated plants and is a characteristic of heat stress effects [14,51]. The decrease in fluorescence can indicate lower electron transport chain (ETC) efficiency at the PS II donor or PS I acceptor side [52]. Additionally, the appearance of the K-band, verified through the significant increase in the K-step amplitude in the non-inoculated individuals, implies PS II oxygen-evolving complex (OEC) damage, representative of low thermo-stability under heatwave conditions [53]. Even though a loss in efficiency in the transport from PQH_2_ to the reduction in PS I final electron acceptors was also noted, an energy flux shift changing PS II / PS I equilibrium towards PS I was observed in the non-inoculated and C1-inoculated plants [54,55] to alleviate excessive energy accumulation at the PS II and attempt redox stability between photosystems. In these same sample groups, heatwave exposure resulted in a significant loss of PS II efficiency in trapping photons, which can be indicative of a potential photoinhibition process most likely due to excess energy absorbed per reaction centre (RC) [56]. Even though the quinone pool size available for oxidation suffered no heat-induced alterations, in non-inoculated grapevines the energy needed to close all reactive centres suffered a significant increase and, as a known counteractive measure, a significant increase occurred in the RC turnover rate and the net rate of PS II reaction centre closure [11,57]. This resulted in excessive absorption of photonic energy unused for chemical energy generation in the ETC; hence, HW-exposed non-inoculated individuals presented a significant increase in their energy dissipation mechanisms, observed as significantly higher leaf temperature (lack of thermal regulation capacity) and photochemical quenching, as seen in the significant increase in the dissipation energy flux (DI/RC) [58,59]. Reaction centre-based energetic parameters corroborate these facts. The photosynthetic apparatus in the non-inoculated plants showed HW-induced energy input instability, as revealed by the significant increase in the absorbed (ABS/RC) and trapped (TR/RC) energy flux; in contrast, transported energy flux (ET/CS) remained unchanged. These results, coupled with the decrease in the fully active RC combined with the increase in heat sink dissipation centres, clearly indicate PS II down-regulation and the potential induction of a photoinhibition state [60]. An overview of the PGPR effects can be seen by analysing the performance index, known for being a sensitive indicator of heat stress that relates energy absorption, antenna steps, reaction centre and electron transport chain functioning [61,62]. As estimated previously, a significantly HW-reduced performance index in the non-inoculated plants was observed when compared to C2-inoculated individuals, which exhibited a performance index increase under heatwave conditions. This higher photochemical performance can be explained by the significantly higher reaction centre II density within the antenna chlorophyll bed and the probability that PS II chlorophyll functions as an RC [63]. The increased reaction centre availability, acquired through C2 consortia inoculation, improved light-harvesting capabilities and allowed the maintenance of the leaf’s photosynthetic efficiency while reducing the photosynthetic antenna size and preventing energetic overload in the RC [26,64]. Photosynthetic stress mitigation can be connected to consortia 1 and 2 rhizobacteria which improved N-fixation capabilities. Extreme temperatures may prime nitrogen dependency in plants, limiting root N assimilation and altering N partitioning among the plant’s components [65,66]. Moreover, nitrogen is a primary component of amino acids, chlorophyll and proteins; substantial amounts are invested in photosynthetic enzymes such as ribulose-1,5-bisphosphate carboxylase (RuBisCo), which are known to be extremely heat sensitive and constitute the early effects of thermal stress [67,68]. The constraints observed in the non-inoculated plants’ ETC may be partially a result of RuBisCo heat-induced inhibition, as described in the studies of [69,70]. Additionally, N-deficiency lowers the PS II protein repair rate and can be related to the degradation of the photodamaged D1 protein, impairing the photochemical apparatus [71,72]. PGPR inoculation had a mitigating heat stress effect, allowing for the accumulation, synthesis and remobilization of nitrogenous-related compounds in grapevine leaves, such as proline, which was found to present control values in inoculated plants exposed to HW treatment. From this, it can be affirmed that PGPR bioaugmentation resulted in a photoprotection enhancement and ETC amelioration of grapevine photosynthetic processes in heat stress conditions, although superior through C2 inoculation. These observations were evidenced in the structure functional indexes. In contrast to the non-inoculated grapevines, although some HW damages were revealed, C1 inoculation allowed for the maintenance of the structural functional index for photosynthesis (SFI) and the functional index for non-photochemical reactions (SFI_NPQ_). However, in comparison to the other plant groups, C2 inoculation showed significantly higher SFI as well as a significantly lower SFI_NPQ_. As both these indexes are intrinsically connected with the overall energy fluxes summarizing the outcome resulting from its balances, it can be deduced that C2-inoculated plants under heatwave exposure were able to maintain a high light energy use efficiency (similar to that observed for its control temperature counterparts), probably promoted by the alleviation of oxidative stress by improved CAT and APx; this feature that was not observed in C1-inoculated plants under heatwave exposure.

The leaf pigment content of *Vitis vinifera*, the de-epoxidation reaction and the carotenoid to chlorophyll ratio showed no HW-induced rise, indicating that biochemical energy dissipation through the xanthophyll cycle was not induced and the photoprotection mechanism was not activated [73]. However, under heatwave conditions, the grapevine was described as using carotenoid-based reactive oxygen species (ROS) scavenging mechanisms [74]. Violaxanthin concentration increased in the bioaugmented groups, in contrast to the decrease in the non-inoculated plants. Nonetheless, the differences in concentration of this efficient energy quencher pigment were not significant [75]. Additionally, all treated groups presented an increase in the β-carotene, zeaxanthin and lutein pigment concentrations, boosting the thylakoid membrane antioxidant potential [76]. Even though some PGPR-induced variation differences were not significant, it is noteworthy that C2-inoculated individuals displayed a comparatively higher concentration of this antioxidant pigment, a possible indication of a comparatively better ROS-savaging capability. This could be provided by the C2 PGPR nitrogen fixation enhancement, phosphate solubilization attribute and iron uptake augmentation by siderophores [77]. Higher accessibility and nutrient uptake from the rhizosphere boost the production of photoreceptor pigments, as indicated by previous studies [23,78]. Moreover, under the control temperature, C1-inoculated plants showed a reduction in the pigment concentrations, yet photosynthetic capability remained the same. 

Under extreme heat stress, leaf osmotic regulation is critically affected [79]. Grapevines appear to maintain leaf water content (WC) even under heatwave conditions, a probable evolutionary adaptation to their Mediterranean native environment. However, inoculation induced WC variation under the control temperature. Under these conditions, C1-inoculated individuals displayed a significantly higher WC, yet no significant differences between groups were observed when HW-exposed. To counteract HW-inducing osmotic pressure, synthesis and accumulation of L-proline in grapevine leaf tissues are generally observed [80]. Accordingly, a significantly higher proline leaf tissue concentration was observed in the non-inoculated plants. In contrast, bioaugmented plants exposed to the heatwave presented the same levels of this osmo-compatible solute as in control conditions. These results indicate a rhizobacteria’s osmoprotectant promotion, alleviating the need for higher osmolyte concentration to maintain leaf turgidity [81]. In addition to its osmoregulation function, proline is vital in maintaining and protecting membrane integrity and ROS detoxification, preventing oxidative bursts in leaves [82,83]. Heat stress increases membrane fluidity, which can cause reaction series disconnection and subsequent changes in metabolism. In plant cells, the uncoupling of reactions combined with the abovementioned photochemical impairment and heightened energy dissipation causes the accumulation of intermediate ROS [84,85]. To alleviate oxidative stress, plants rely on enzyme-based antioxidant mechanisms to preserve redox homeostasis [86]. Considering the oxidative stress biomarkers, rhizobacterial inoculation resulted in group-specific oxidative stress response variation, evidenced in the canonical analyses’ (CAP) high classification efficiency. First, C1 inoculation under normal temperature stimulated a comparative increase in superoxide dismutase (SOD), guaiacol peroxidase (GPx) and ascorbate peroxidase (APx) activities in a way that led to a significantly higher oxidative ratio of one as compared to their counterparts. This suggests a hydrogen peroxide generation/decomposition equilibrium [87]. This can imply a C1-induced carotenoid–ROS-quenching strategy in reducing hydrogen peroxide from other sources following the abovementioned total carotenoid loss [88]. Furthermore, when comparing HW-treated grapevines, C1-inoculated plants displayed a significantly higher oxidative ratio, possibly due to deficiencies in catalase (CAT) and GPx activities. Nonetheless, when observing the lipid peroxidation products (TBARS) present in the HW-exposed leaves, only non-inoculated individuals showed a significant increase. At the same time, PGPR consortia-treated plants were able to maintain non-stressed levels. The increase in lipid peroxidation suggests that non-inoculated grapevines’ antioxidant mechanisms were insufficient to endure the heatwave-related oxidative stress, resulting in physiological damage within plant cells [13,89] and keeping within the noted photosynthetic efficiency loss. Fatty acids displayed membrane protection mechanisms to reduce cellular membrane degradation by ROS toxicity and excessive fluidity due to high temperatures. The significant increase in the monounsaturated fatty acid (MUFA) concentration, coupled with the significant reduction detected in the polyunsaturated fatty acids (PUFA) and in the double bound index (DBI) of both non-inoculated and C1-inoculated plants, are indicative of membrane remodelling, reflecting a decrease in membrane fluidity [55]. In these same groups, a significant reduction in linolenic acid (C18:3) content can also suggest their utilization as a direct ROS scavenger, concomitant with the TBARS accumulation in non-inoculated plants and the low antioxidative enzyme present in the C1-inoculated individuals [59]. It can be noted that the C2-inoculated group showed no significant changes in the FA saturation classes’ relative abundance changes when exposed to thermal treatments. Grapevine exhibited a significative reduction in oxidative and membrane damage under heatwave conditions by consortium 2 inoculation in concordance with previous PGPR studies [35,90,91]. All this connected with an improved thermotolerance can be at the basis of the inoculated plants’ resilience to the tested HW treatment.

Multivariate analysis delivered an integrated approach and overview of PGPR consortia’s influence on the biophysical and biochemical traits of *V. vinifera*. These allow an understanding of the metabolic pathways that exhibited significant PGPR contribution. When observing the canonical analyses, oxidative stress biomarkers were the most effective in distributing all grapevine groups; this indicates significant PGPR-induced trait modification in both thermal treatments, probably due to stress alleviation through plant internal ethylene concentration reduction. Nonetheless, the other highlighted datasets exhibited meaningful insights. As mentioned previously, C1-inoculated plants displayed a specific and distinguished pigment profile under normal conditions. Moreover, C2-inoculated plants, when HW-exposed, displayed a clearly defined grouping in the canonical space of the photochemical processes and comparatively better separation in the pigment and fatty acid profiles. This overview emphasizes the evident photochemical and physiological bioaugmentation impact, as well as the contrast of the action of the different consortia. In summary, *Vitis vinifera*, when inoculated with the PGPR consortia, had a significant amelioration effect on heatwave-induced stress. These consortia were designed with tested and proven plant growth-promoting rhizobacteria that presented great bioinoculant potential during in vitro assays [27,28] and were built with no differentiating factor among them; however, superior oxidative protection and overall fitness were seen in consortium 2. Consortia effectiveness variation can have many explanations, including biotic and abiotic stress sensitivity [92], soil biochemistry [93] and leaf surface biochemistry [94]. Bacteria selection and consortia design is a key determinant of the effectiveness of the bacterial inoculant and the finished product itself. Thus, a knowledge-based solution for this formulation or an experience repository is being debated to maximize research efficacy [95]. Furthermore, long-term ecological implications for the use of allochthonous bacteria as agro-inoculants have not been reported. Most of the authors that developed field trials with bioinoculants observed that they have temporary effects on soil and plant growth. For example, PGPR used in sunflower, tomato, pigeon pea and maize showed an inoculation effect at the early stages of plant growth, but it decreased at later stages. They also showed initial re-shaping of soil bacterial communities after inoculation that was limited to rhizospheric microbiota at later stages [96,97]. These results suggest that the survival and proliferation of allochthonous microorganisms in soil are limited. This was confirmed by [98], who detected the inoculated strain approximately two months after sowing. Hence, determining the concentration of inoculum formulations and their presence in soil over time, as well as optimizing biofertilizer application through irrigation or other methods, is necessary before delivery to the agricultural market. Additional research relating to transcriptomic and metabolomic analyses is planned and required to gain further insights into the role of PGPR in stress protection.

## 5. Conclusions

Marine rhizobacteria-bioaugmented grapevine plants exhibited significantly superior photoprotection and membrane stability and displayed a notable amelioration in the oxidative stress experienced when exposed to a heatwave stress. Therefore, compared to the heat-stressed non-inoculated grapevines, an overall PGPR-induced improvement of plant fitness was witnessed. This approach also found that even though selected bacterial strain combinations revealed similar potential as bioinoculant in in vitro assays, the application in this study presented significant differences in the heatwave amelioration effectiveness between the tested consortia. It can be inferred that grapevine, when inoculated with consortium 1, presented heatwave stress tolerance (stress avoidance); however, when inoculated with consortium 2, it presented heatwave amelioration (increased resistance). On this basis, the PGPR consortium constituted by *A. aquariorum*, *B. methylotrophicus* and *B. aryabhattai* can be a remarkable and viable tool for mitigating and avoiding heat-related harm in *Vitis vinifera*. These findings illustrate the potential of PGPR bioaugmentation and the possible biotechnological capabilities for mitigating climate change’s harmful effects on agricultural productivity; nevertheless, the mechanism of how PGPR imparts abiotic stress tolerance is still not completely understood. Research is needed to depict intrinsic effectiveness linked to bacteria–plant and bacteria–stress relations for role-specific consortia formulation. Developing PGPR consortia designs particular to individual species and expected stress impositions is possible, thus highlighting the applicability of PGP rhizosphere engineering and the importance of further investigation.

## Figures and Tables

**Table 2:** Relative concentrations of pigments (µg g^−1^ FW): chlorophyll *a* (Chl *a*), chlorophyll *b* (Chl *b*), total chlorophyll (TChl), pheophytin *a* (Pheo *a*), total carotenoid (Tcarot), β-carotenoids (βcarot), antheraxanthin (Anthe), lutein, violaxanthin (Viola) and zeaxanthin (Zea); de-epoxidation state (DES), chlorophyll *a*/*b* ratio (Chl *a/b*), total carotenoid to total chlorophyll ratio (TCar/TChl) and chlorophyll degradation index (CDI), in non-inoculated (−I) and marine PGPR consortia 1- and 2- inoculated (+I) *Vitis vinifera*, under normal (control) thermal regimes and exposed to the heatwave (HW) (average ± standard deviation, N = 5, letter denotes significant differences at *p* < 0.05).

		Chl *a*	Chl *b*	TChl	Pheo *a*	Tcarot	βcarot	Anthe	Lutein	Viola	Zea	DES	Chl *a*/*b*	Tcar/TChl	CDI
C	−I	851.8 ± 159.4 ^bc^	367.1 ± 130.1 ^ab^	1219.0 ± 286.1 ^bc^	147.1 ± 105.1 ^a^	359.6 ± 62.9 ^ab^	51.1 ± 9.4 ^ab^	42.3 ± 7.53 ^a^	62.6 ± 9.1 ^ab^	61.0 ± 14.8 ^a^	114.0 ± 38.3 ^ab^	0.482 ± 0.065 ^a^	2.420 ± 0.364 ^ab^	0.298 ± 0.018 ^a^	0.163 ± 0.082 ^a^
	+I1	702.4 ± 131.0 ^c^	261.0 ± 47.0 ^b^	963.4 ± 172.1 ^c^	104.9 ± 56.7 ^a^	255.7 ± 35.4 ^b^	38.5 ± 9.4 ^b^	28.7 ± 9.93 ^a^	52.4 ± 10.8 ^b^	32.8 ± 12.3 ^a^	56.6 ± 9.9 ^b^	0.519 ± 0.071 ^a^	2.700 ± 0.267 ^a^	0.268 ± 0.024 ^a^	0.129 ± 0.058 ^a^
	+I2	923.2 ± 140.5 ^abc^	340.1 ± 80.2 ^ab^	1263.3 ± 220.5 ^abc^	70.6 ± 37.5 ^a^	363.9 ± 75.3 ^ab^	46.7 ± 7.0 ^ab^	44.3 ± 6.43 ^a^	66.7 ± 13.2 ^ab^	58.0 ± 32.8 ^a^	119.2 ± 28.5 ^ab^	0.452 ± 0.068 ^a^	2.756 ± 0.228 ^a^	0.287 ± 0.012 ^ab^	0.070 ± 0.028 ^a^
HW	−I	1083.3 ± 287.6 ^ab^	492.0 ± 127.7 ^a^	1575.3 ± 413.3 ^ab^	151.8 ± 72.6 ^a^	445.4 ± 112.0 ^a^	55.5 ± 11.6 ^ab^	45.9 ± 9.33 ^a^	77.9 ± 26.3 ^ab^	56.6 ± 30.5 ^a^	165.1 ± 25.4 ^a^	0.374 ± 0.062 ^a^	2.203 ± 0.136 ^b^	0.284 ± 0.024 ^ab^	0.119 ± 0.048 ^a^
	+I1	1024.9 ± 65.2 ^ab^	505.7 ± 46.3 ^a^	1530.5 ± 89.5 ^ab^	152.1 ± 32.2 ^a^	396.1 ± 33.3 ^a^	44.4 ± 9.2 ^ab^	40.9 ± 6.03 ^a^	77.3 ± 11.7 ^ab^	71.2 ± 25.4 ^a^	137.7 ± 26.6 ^a^	0.447 ± 0.080 ^a^	2.039 ± 0.215 ^b^	0.259 ± 0.012 ^ab^	0.129 ± 0.026 ^a^
	+I2	1325.7 ± 273.5 ^a^	554.5 ± 144.2 ^a^	1880.2 ± 417.2 ^a^	132.8 ± 25.1 ^a^	461.4 ± 95.5 ^a^	56.7 ± 11.3 ^a^	48.3 ± 9.33 ^a^	90.4 ± 22.73 ^a^	76.7 ± 27.0 ^a^	165.9 ± 42.2 ^a^	0.427 ± 0.046 ^a^	2.417 ± 0.131 ^ab^	0.246 ± 0.010 ^c^	0.093 ± 0.017 ^a^

## Data Availability

Data available upon request.

## Supplementary Materials

The following supporting information can be downloaded at: https://www.mdpi.com/article/10.3390/microorganisms11040856/s1, Table S1: Summary of fluorometric analysis parameters and their description.
