# Supplementary material for: Improving Grapevine Heat Stress Resilience with Marine Plant Growth-Promoting Rhizobacteria Consortia
Source: Microorganisms. 2023 Mar 27;11(4):856. doi: 10.3390/microorganisms11040856 (PMC10141645; doi:10.3390/microorganisms11040856)
Supplement: Supplementary file 1 [file microorganisms-11-00856-s001.zip › microorganisms-2222628-supplementary.pdf]

Supplementary Table S1. Summary of fluorometric analysis parameters and their description.

Fv/Fm	Photosystem II (PS II) quantum yield.
Area	Area above the Kaustky curve, proportional to the size of the oxidized quinone pool.
N	Quinone redox turnover rate.
γRC	The probability that a chlorophyll *a* molecule functions as a light-harvesting reaction centre.
δR_0	The efficiency of the transfer of an electron from PQH_2 to final PS I acceptors.
M_0	The net rate of PS II RC closure.
SM	Corresponds to the energy needed to close all reaction centers.
V_K	Relative variable fluorescence K-step.
V_J	Relative variable fluorescence J-step.
K-band amplitude	The amplitude of the K-step ($W_K = V_K - V_J$).
$\psi_{E0}/(1 - \psi_{E0})$	The equilibrium constant for the redox reactions between PS II and PS I
PI	Performance index on absorption basis, incorporating the steps of antenna, reaction centre and electron transport parameters.
RC/ABS	Reaction centre II density within the antenna chlorophyll bed of PS II
SFI	Structure functional index for photosynthesis.
SFI (NPQ)	Non-photosynthetic or dissipation structure functional index.
ABS/RC	Absorbed energy flux by reaction centre (RC).
TR/RC	Trapped energy flux by reaction centre (RC).
ET/RC	Electron transport energy flux by reaction centre (RC).
DI/RC	Dissipated energy flux by reaction centre (RC).
RC/CS	The number of available reaction centres per cross-section.
